# Self-Employment in Later Life: How Future Time Perspective and Social Support Influence Self-Employment Interest

**DOI:** 10.3389/fpsyg.2019.00448

**Published:** 2019-03-04

**Authors:** Valerie Caines, Joanne Kaa Earl, Prashant Bordia

**Affiliations:** ^1^Flinders Business, College of Business, Government and Law, Flinders University, Adelaide, SA, Australia; ^2^Department of Psychology, Faculty of Human Sciences, Macquarie University, Sydney, NSW, Australia; ^3^Research School of Management, Australian National University, Canberra, ACT, Australia

**Keywords:** social cognitive career theory, social support, future time perspective, self-employment, older workers, entrepreneurship

## Abstract

For older workers, self-employment is an important alternative to waged employment. Drawing on social learning theory and social cognitive career theory we examine how attitudes toward one’s own aging, future time perspective (captured by perceived time left to live) and perceived support from referent individuals predict self-efficacy for entrepreneurship and outcome expectations, influencing self-employment interest. Findings from a sample of professional association members (*n* = 174, mean age 52.5 years), revealed that an open-ended time perspective relates positively to entrepreneurial self-efficacy, while social support relates positively to outcome expectations. Consistent with social cognitive career theory, entrepreneurial self-efficacy mediated the relationship between future time perspective and interest in self-employment, and outcome expectations mediated the relationship between social support and interest in self-employment. This study extends current career and entrepreneurship theory in several ways. First, the inclusion of age-related psychosocial and sociocultural factors in the study model shed light on the intersection between older age, the contextual environment and development of self-employment interest. Second, the findings support earlier arguments that older entrepreneurship is a social process whereby the social context in which people work and live influences their interest in entrepreneurship, and that entrepreneurial behavior among older people needs to be supported to occur. Finally, the findings suggest the utility of social cognitive career theory in informing the development of self-employment interest in the late career stage. We discuss implications for the career and entrepreneurship literatures as well as practitioners involved in late-career counseling or seeking to promote entrepreneurship for older people.

## Introduction

Older workers will become an ever-increasing segment of the workforce in developed countries with many older workers expected to work beyond ‘normal’ retirement age (Maritz, 2015; Kautonen et al., 2017; Stirzaker and Galloway, 2017). Consequently, workforce aging has become a policy focus driven by the efforts to delay retirement and dependency on government resources. With the emphasis on working longer, older individuals are increasingly seeking sustainable career options. However, the concept of a second or even third career is novel, as such we have an incomplete understanding of how older workers formulate career interests and goals.

Late career is widely conceptualized as the period prior to retirement, commencing when a worker is around 50 years of age and mid-career as the period from 35 to 50 years of age (Van der Horst et al., 2016; Kautonen et al., 2017). Individuals are likely to develop interests and goals for their late-career from late in their mid-career onwards (approximately 45 years of age onwards). This time also coincides with the age at which workers begin to perceive themselves as ‘older workers’ in part because of increased difficulty obtaining employment, experiences of age-related biases and discrimination, and the awareness of the onset of physical and cognitive decline (Curran and Blackburn, 2001; Kibler et al., 2012).

The idea of working longer challenges our understanding of ‘career’, commonly conceptualized as the ‘sequential, predictable, organized path through which individuals pass at various stages of their working lives’ (Holmes and Cartwright, 1993, p. 37) ending in full withdrawal from the workforce. This conceptualization of career does not reflect contemporary working life (van Loo, 2011). More than ever careers are unlikely to be sequential, predictable or hierarchical in nature (Holmes and Cartwright, 1993; Voelpel et al., 2012); individuals are likely to switch job and occupations in what is often described as a ‘boundaryless career’ (Arthur, 1994; Sullivan and Emerson, 2000). Additionally, a contemporary career requires individuals to actively self-manage their career journey rather than be passive participates in a well-defined career path (Hall, 1996; van Loo, 2011).

Elements of the contemporary career are evident among older workers who may transition careers, move in and out of retirement and move from paid employment to entrepreneurship (Alcover et al., 2014). Notably, self-employment is promoted as a means for older individuals to delay retirement or create employment for themselves (Singh and DeNoble, 2003; Kautonen et al., 2017). The attitudes and motivations of older people who take up self-employment has not been extensively researched. The available research suggests that the motivations of older entrepreneurs may differ from younger cohorts embarking on an entrepreneurial career path. For example, older workers may be responding to negative experiences in corporate life, such as age-related discrimination and job loss (Curran and Blackburn, 2001; Kibler et al., 2012), or seeking an income for themselves, on their terms. However, it is also apparent that being enterprising in later life is inconsistent with the accepted narrative regarding aging at work, which is focused on withdrawal and decline (Ainsworth and Hardy, 2008). This narrative suggests that older individuals may be deterred from making enterprising career choices and may be unsupported.

The research concerning older entrepreneurs is sparse (Wang and Shi, 2014; Zolin, 2015, p. 36; Gielnik et al., 2018) which is not unexpected given its emergent nature. Previous researches have identified how the choice of an entrepreneurial career intersects with diversity markers such as gender and ethnicity (Wheadon and Duval-Couetil, 2017; Griffin-EL and Olabisi, 2018). However, there has been limited research on how entrepreneurship intersects with age, specifically old age (Ainsworth and Hardy, 2008; De Kok et al., 2010). Older individuals have been included in the advancement of ‘inclusive entrepreneurship’ along with other minority groups in parts of Europe (Pilkova et al., 2014). However, age is arguably a unique dimension of diversity as everyone will eventually become older, while age-related markers are almost impossible to hide (Ainsworth and Hardy, 2008).

The literature identifies several personal and background factors which may well differentiate older people becoming first time entrepreneurs from younger cohorts, such as extended time for skill development (Davidsson and Honig, 2003; Ahmad et al., 2012), work and life experience, maturity, and wisdom (Botham and Graves, 2009; Gordon and Jordan, 2017), favorable financial status (Kibler et al., 2012; Stirzaker and Galloway, 2017), expansive career and social networks (Davidsson and Honig, 2003; Ahmad et al., 2012) and age-related health concerns (Curran and Blackburn, 2001; Radford et al., 2015). Additionally, with increasing age comes the challenge of diminishing available time, complex social roles (i.e., caring responsibilities) and navigating the influence of age-related stereotypes and prejudices (Kautonen et al., 2011; Kibler et al., 2012).

The entrepreneurial environment may also play a salient role in determining the level of older entrepreneurship. Clarke and Holt (2010) argue that coming up with a novel business idea is not enough for a new venture to be successful, the ideas must also be publicly acknowledged and supported. There is evidence suggesting that cultures which accept seniors have a strong positive influence on the incidence of older entrepreneurship (Weber and Schaper, 2004; Zhang, 2008). However, there is also evidence to suggest that older entrepreneurs may be socially excluded. For instance, Kibler et al. (2015) concluded that if entrepreneurship in later life is to be cultivated there will need to be increased awareness of potential age-related discrimination and strategies developed for managing these. Furthermore, the social discourse regarding older workers in an enterprising context is principally negative and reinforces many of the stereotypes of older people as workers in general. As entrepreneurs, the discourse suggests that “older workers make bad consumer decisions, e.g., buy a business on impulse” and “as entrepreneurs are ‘a risky project,’ they want too much safety and security, and they take irresponsible risks” (Ainsworth and Hardy, 2008, p. 395). Consequently, the relationship between older age and entrepreneurship remains unclear and is both interesting and important from a career choice and entrepreneurship perspective.

Anchoring in the social cognitive career theory (SCCT) framework, this study makes a contribution to our understanding of how late career interest develops, more specifically interest in self-employment, in an overlooked group (older workers), adding to the growing body of recent SCCT research among older workers (Wöhrmann et al., 2013; Garcia et al., 2014). Additionally, although older workers are often contextualized as a homogenous group, with a focus on chronological age, the identification of age-specific background and personal factors which influence late career choice suggest that career interest development in later life is complex and multi-faceted. As such, late-career decisions are dynamic and idiosyncratic adding support to the emerging body of career research suggesting that older workers are heterogeneous (Sterns and Miklos, 1995; Bal and Jansen, 2015) and will require individual late career working arrangements that can meet each individual’s motivations and needs.

## Theory and Hypotheses

The intention to become self-employed can be conceptualized as a career choice, which we argue is compatible within the explanatory scope of SCCT (Lent et al., 1994). While SCCT has been predominantly applied to career choice, more recently it has been successfully applied to understand the formation of entrepreneurial intentions and career adaptability across the lifespan (Lanero et al., 2016; Lent et al., 2016, 2017).

**FIGURE 1:**
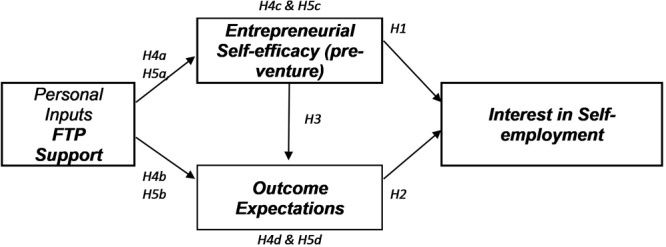
Conceptual model of self-employment interest in older workers.

Within the SCCT framework, Lent et al. (1994) propose a model explaining how career interests are developed overtime, influenced by cognitive and behavior factors. Figure 1 depicts our conceptual model. The formation of interest (i.e., likes, dislikes and disinterests toward a career or occupation) is hypothesized by Lent et al. (1994) to be an antecedent to career choice. When applied to self-employment the SCCT interest model hypothesizes that before any entrepreneurial-related activity is commenced individuals go through a preparatory phase, where interest emerges. This preparatory phase is also alluded to in the entrepreneurship literature as a conception and gestation stage which occurs before any action is taken to start a business (Aldrich and Martinez, 2001), although there has been little research examining this stage. Studies which have explored the pre-venture stage have frequently studied nascent entrepreneurs or individuals already involved in starting a new venture (Soutaris et al., 2007), which only provides limited insight on how interest emerges.

In the SCCT interest model, Lent et al. (1994) hypothesizes that perceptions of self-efficacy and outcome expectations predict career interest. Self-efficacy is an individual’s belief that they can do a particular task, is developed over time (Bandura, 1982), and influenced by various learning experiences: enactive attainment; accomplishments; vicarious experiences (observational learning, modeling, and verbal persuasion); and an individual’s psychological state (Bandura, 1986). The nature and availability of learning experiences is influenced by background and personal factors. For example, being older may restrict what learning experiences are accessed, as well as the support and feedback received. Repeated performance accomplishments and mastery experiences are considered the most effective way to develop efficacy beliefs (Bandura, 1977, 1982; Wood and Bandura, 1989; Scherer et al., 1991). While vicarious learning is argued to be less effective for developing efficacy beliefs capable role models can affect self-efficacy through social comparison (Gist, 1987; Wood and Bandura, 1989). For example, older individuals who are successful in the career or self-employment domain can lead other older individuals to believe they can be successful too.

In addition to self-efficacy, Lent et al. (1994) hypothesize that the beliefs an individual holds about what the outcome response might be from performing a particular behavior influence the development of career interests. Further, outcome expectations are predicted to be partially influenced by self-efficacy as individuals will anticipate a more positive outcome if they have the belief they will succeed and can predict success (Wöhrmann et al., 2013).

There is a significant body of research which has identified self-efficacy and outcome expectations as important predictors of interests in both the career and entrepreneurial context (Segal et al., 2005; Zhao et al., 2005; Drnovšek et al., 2010; Lent et al., 2010; Sheu et al., 2010; De Clercq et al., 2013; Mortan et al., 2014; Tsai et al., 2014). Self-efficacy for a career domain gives people a sense of confidence and motivates them to work toward careers they perceive as attainable. However, Lent et al. (1994) also argues that the influence of self-efficacy and outcome expectations (individual or together) on behavior will depend on the type of behavior. Entrepreneurship is a high-risk endeavor. Hence, in the case of costly decisions SCCT proposes that both self-efficacy and outcome expectations influence interest directly. For instance, an individual with high self-efficacy for entrepreneurship would not develop an enduring interest if they anticipated a negative outcome (e.g., non-support of referent others, conflict, or financial loss).

### Entrepreneurial Self-Efficacy and Outcome Expectations

Self-efficacy for entrepreneurship is characterized by entrepreneurial self-efficacy (ESE). ESE is commonly described as the confidence of entrepreneurs to undertake specific tasks in the entrepreneurial domain or confidence in the personal ability to realize the business start-up process (Chen et al., 1998; Segal et al., 2005). The influence of self-efficacy in the development of entrepreneurial intentions has primarily been examined with young college students. Nevertheless, there is strong empirical support for the argument that individuals with high ESE are more likely to be interested in entrepreneurship (Chen et al., 1998; Zhao et al., 2005), and take steps to become entrepreneurs (Townsend et al., 2010). Therefore, we expect that self-efficacy for the tasks related to entrepreneurship helps explain the development of entrepreneurial interest among older workers; those with higher levels of ESE will be more likely to express an interest in self-employment. Thus, it is predicted that:

H1: Entrepreneurial self-efficacy will be positively related to interest in self-employment.

In addition to ESE, SCCT predicts that outcome expectations —OE will also influence self-employment interest. OE differs from self-efficacy, which is a belief about being able to do something, as it involves the imagined consequences of performing a behavior. Bandura (1986) suggested three types of OE: physical, such as money; social, such as approval; and self-evaluation, leading to satisfaction. In the context of this study, OE refer to expectations about the outcome from self-employment; for example, an individual might expect the outcome from starting a business to be job satisfaction, skill development or financial reward. In both the career and entrepreneurship literature, there has much less emphasis on outcome expectations as the determinant of career interest or action (De Clercq et al., 2013; Lanero et al., 2016). However, SCCT points out that an individual will act on their beliefs of what they can do [self-efficacy], as well as on their expectations regarding the likely consequences of those actions (Bandura, 1986; Lent et al., 2010). Consequently, we expected that OE are related to self-employment interest. Thus, it is predicted that:

H2: Outcome expectations will be positively related to interest in self-employment.

Social cognitive career theory also predicts that self-efficacy causally influences outcome expectations (Bandura, 1986). Additionally, in the career literature self-efficacy has been shown to predict career choice goals both directly and through outcome expectations (Sheu et al., 2010). For instance, individuals who feel efficacious about an activity are more likely to also anticipate a positive outcome from undertaking that activity. Bandura (1986) further predicts that self-efficacy is the more influential determinant. Additionally, Searle (2001) argues that an individual’s consciousness of their personal capability forms the foundation of human action. Where an individual does not perceive that they have the capability to undertake an action, they are unlikely to do so. Therefore, an older worker may believe that self-employment in later life would be a viable way to prolong their career and increase their income, but do not pursue the idea because they doubt their ability to start a business venture. Thus, it is predicted that:

H3: Entrepreneurial self-efficacy will influence outcome expectations.

### Future Time Perspective and Self-Employment Interest Mediated by Entrepreneurial Self-Efficacy and Outcome Expectations

The nature of entrepreneurship requires the entrepreneur to develop and implement goals and plans which require a cognizance of and purpose for the future. Having goals for the future motivates individuals to achieve long-term goals which they value (McInerney, 2004). However, psychologists in the clinical and gerontology disciplines have for some time noticed age-related differences in how people engage in social activity and goal setting (Carstensen, 1993, 1995, 2006; Cate and Oliver, 2007).

One approach to explaining age-related differences in social behavior and goal setting is drawn from socioemotional selectivity theory (SST) which is grounded in lifespan theory (Carstensen, 1993, 1995, 2006; Carstensen et al., 1999; Lang and Carstensen, 2002; Carstensen and Lang, Unpublished). SST predicts the changes in social behavior across three social motives – emotional regulation, self-concept, and information seeking (Carstensen, 1993). While each of the social motives is present throughout the lifespan, it is argued that their salience changes over time (Carstensen, 1995). Specifically, individuals select their goals based on their perceptions of whether time is limited or open-ended referred to as future time perspective – FTP (Carstensen et al., 1999; Lang and Carstensen, 2002; Carstensen and Lang, Unpublished). Put simply, FTP refers to how much time individuals’ perceive they have left to live (Cate and Oliver, 2007). This is quantified as time left being perceived as *limited or expanded (open-ended)* (Carstensen, 1993; Carstensen et al., 1999). When time is perceived as limited individuals are more likely to focus on short-term and emotionally meaningful goals such as emotion regulation or generative needs (Lang and Carstensen, 2002). In contrast, when time is perceived as open-ended individuals focus on longer-term goals including information seeking (Carstensen, 1995) and knowledge-related goals (Lang and Carstensen, 2002).

Future time perspective has been the focus of several studies examining work motivation (Sonnentag, 2012; Strauss et al., 2012). There has also been several small studies exploring the relationship between FTP and financial planning for retirement, retirement adjustment (Van Solinge and Henkens, 2009; Yang and Devaney, 2011; Griffin et al., 2012), and entrepreneurship (Gielnik et al., 2018). The results reveal how FTP influences long term goal setting and motivation. For instance, individuals with an expanded FTP preferred to retire later (Van Solinge and Henkens, 2009) and are less likely to plan financially for retirement (Yang and Devaney, 2011).

Consequently, SST is a useful theory to incorporate into the present research for two reasons. Firstly, SST has demonstrated empirically that social changes in later life are not only determined by chronological age but also cognitive and motivational changes (Carstensen, 1993). Secondly, the relevance of SST to this study comes from the understanding that career choices are made with a conscious or unconscious awareness that time is limited or open-ended. As a consequence FTP integrates the anticipated future into the present time (Seginer and Lens, 2015) and therefore is complimentary to a social cognitive model of career interest for older individuals (Zacher and Frese, 2009).

Future time perspective provides a useful lens to understand how age differences impact career choice as a consequence of an individual’s perception of the time they have left to live, including the assessment of opportunities and goals available within that time (Carstensen, 1995; Lang and Carstensen, 2002). There are considerable differences in how much time older people believe they have left to live (Zacher and Frese, 2009). Fredrickson and Carstensen (1990) found that regardless of age where an individual imagines conditions outside of the normal life span (i.e., older person imagining an expanded future time or younger person imaging a restricted future time) chronological age differences in goal choice disappear. Therefore, time perspective may be more useful than chronological age in predicting social motivations and goals, including career choice.

Open-ended and limited time perspectives are distinguished by their differential effect on work motives (Carstensen, 1995; Kooij and Van De Voorde, 2011; Kooij et al., 2011) and are likely to also influence interest in self-employment in older individuals. A future orientation, enacted by a willingness to network, and seeking knowledge are closely aligned to the pre-venture dimensions of ESE. For instance, older workers with an expanded FTP may be more motivated to develop social relationships which are oriented toward future benefits and knowledge acquisition. Additionally, an expanded FTP may influence the assessment of extrinsic and intrinsic outcomes (gains and losses) from self-employment (Carstensen, 1995). For instance, an individual with an expanded FTP may perceive starting a business as an opportunity to gain future wealth and satisfaction rather than focusing on short-term risks such as the potential loss of having a failed business. More specifically, we hypothesize that older workers with an open-ended FTP will be more likely focus on their long-term career and are therefore more likely to be interested in prolonging their career through self-employment. Conversely, employees with a limited FTP will be more likely to focus on short-term positive emotions and retirement. Additionally, individuals with a more expanded FTP may perceive more favorable outcomes from self-employment than those with a limited FTP. Thus, it is predicted that:

H4a: FTP will be positively related to ESE.
H4b: FTP will be positively related to OE.

Further, in SCCT personal and background factors are theorized to indirectly influence the development of interest. Interest is argued by Lent et al. (1994) to be principally influenced by perceptions of self-efficacy and anticipated outcome expectations. Therefore, consistent with SCCT it is expected that the relationship between future time perspective and interest in self-employment will be mediated by ESE and outcome expectations. Thus, it is predicted that:

H4c: The relationship between FTP and interest in self-employment is mediated by ESE.
H4d: The relationship between FTP and interest in self-employment is mediated by outcome expectations.

### Social Support and Self-Employment Interest Mediated by Entrepreneurial Self-Efficacy and Outcome Expectations

Theoretically, we argue that social support will act as a background affordance (Lent et al., 2003; Lent, 2005) indirectly influencing the development of self-employment interest. Differences in individual socialization such as verbal encouragement, role models, stereotypes, family values and anticipated approval can influence self-efficacy and outcome expectations (Hackett and Betz, 1981; Lent et al., 1994). For instance, role models, in addition to providing a referent for social comparison may also be sources of support through the provision of feedback and information (BarNir et al., 2011). Support provided in the form of positive feedback, approval and encouragement can influence self-efficacy beliefs (Bandura, 1986) by convincing an individual they can perform a task. Consequently, positive feedback and praise enhances self-efficacy (Bandura, 1977, 1986) while negative feedback decreases self-efficacy and outcome expectations.

The role of social support in the development of older workers’ entrepreneurial career choice has become of interest to researchers seeking to understand the influence of the social context in which enterprises start. Researchers argue the importance of looking beyond the transaction of buying or creating a new venture to include the potential entrepreneur’s social context (Weber and Schaper, 2004; Wainwright et al., 2011). The social network of older workers comprises four salient groups; (1) life partner (spouse); (2) family; (3) friends; and (4) work colleagues. Social groups may have different levels of salience over the various stages of venture creation. For instance, during the early stages, the *motivation* stage, individuals will discuss their ideas with a small group of close contacts – possibly just close friends and family (Greve and Salaff, 2003). They are unlikely to announce their ideas publicly at this stage as that would make it hard to change course.

Prior research examining the influence of social support on entrepreneurial intentions suggest having a spouse has been linked positively to entrepreneurial activity (Özcan, 2011) however, why this is the case is unclear. Family and friends are also cited in the literature as sources of support for starting a business. However, the type and degree of support may vary. For instance, studies have shown that when families have entrepreneurial experience, they were encouraging of their older family member entering self-employment (Davidsson and Honig, 2003; Kibler et al., 2012). In contrast, if the families had no prior experience of entrepreneurship they were opposing and less supportive. There are several studies which argue that family support is critical to starting a new venture (Dyer, 1994; Dyer and Handler, 1994; Davidsson and Honig, 2003). For instance, Greve and Salaff (2003) found that family support was significant for women, who utilize family as a referent point to a much larger extent than men. The evidence is similar for support from friends. For instance, Wainwright et al. (2011) found that older people whose friends had a professional or corporate career regarded being self-employed at an older age as abnormal and inconsistent with their expectations about older professionals. In contrast, where an older person had a friendship group that included entrepreneurs, they perceived more support. The importance of social support is further evidenced by Davidsson and Honig (2003) who observe that social networks are more salient than contact with government agencies in achieving business start-up.

Perception of community support also influence the uptake of entrepreneurship among older people. For instance, Kautonen et al. (2011) found that when an older person perceived that the community was open-minded to older people being self-employed, this positively influenced their entrepreneurial intentions. This finding suggests that if the community accepts that older people can be successful as entrepreneurs this increases older individual’s expectation of a positive outcome from entrepreneurship. Therefore, support may act as an antecedent to outcome expectations. The importance of support is also noted in the retirement literature which concludes that social support assists older workers to continue working (Flynn, 2010). Likewise, the absence of support from referent individuals may be a barrier to the development of interest in self-employment (Ainsworth and Hardy, 2008).

In SCCT it is argued that support is a dimension of learning experiences which influence the development of self-efficacy and outcome expectations (Lent et al., 1994). It is therefore expected, consistent with SCCT and the entrepreneurship intention literature, that partners, family and friends will play highly influential roles, particularly in providing support at the interest development stage (Lent et al., 1994; Kautonen et al., 2009, 2010, 2011; Kibler et al., 2012). The role of work colleagues as referent individuals in career choice or self-employment has not been widely researched. Casey (2009) points out that in Japan many older workers transition to self-employment with the support and approval of their organizations. We therefore also include colleague support in our model’s conceptualization of referent support.

As discussed earlier, in SCCT personal and background factors are theorized to indirectly influence the development of interest. Therefore, consistent with SCCT it is expected that relationship between support and interest in self-employment will be mediated by ESE and outcome expectations. Thus, it is predicted that:

H5a: Support will be positively related to entrepreneurial self-efficacy.
H5b: Support will be positively related to outcome expectations.
H5c: The relationship between support and interest in self-employment is mediated by ESE.
H5d: The relationship between support and interest in self-employment is mediated by OE.

## Materials and Methods

### Sample and Procedure

We used an online survey design to collect information from participants. The participants for this study came from the membership of a professional association in Australia, the first author’s professional networks and referrals from participants (snowballing). Individuals were invited to participate in the study via email and those interested clicked on a link provided in that email to the on-line survey. Respondents first read the participant information statement before providing informed consent (on-line) to proceed with participation. We received 186 responses. Further, 12 responses were omitted due to missing question responses. The final sample size comprised 174 people aged between 40 and 78 years of age. The mean age of participants was 52.5 years (*SD* = 7.16). There were slightly more females (*n* = 93, 53.4%) than males (*n* = 81, 46.6%), with males slightly older than females [*M* = 55.19, 51.41; *t*(172) = 3.58, *p* < 0.001, two-tailed]. A total of 139 (79.9%) participants were married or living as a couple, and 81.2% (138) reported that they had at least one person they were responsible for financially. Some 70 (40%) participants held a postgraduate degree, 27 (15.5%) had a graduate certificate/diploma, 32 (18.4%) had a bachelor’s degree, 27 (15.5%) had an advanced diploma or diploma, 10 (5.7%) had a certificate, and 8 (4.6%) had completed secondary school. Most participants were in ongoing employment (*n* = 133, 76.4%), with the remainder in contingent (casual or contract) roles. Eighty-two (47.1%) participants identified themselves as executive/managers, 76 (43.7%) as professionals, 9 (5.2%) as clerical/administrative worker, 2 (1.1%) as technician/trade worker, 1 (0.6%) as a sales worker, 3 (1.7%) as community and personal workers, and 1 (0.6%) as a laborer. One hundred and eighteen (67.8%) participants were from the private sector, and 56 (32.2%) were from the public sector. The length of time with their current employer ranged from less than 1 year to 46 years, with a mean tenure of 10.5 years.

#### Ethics Statement

In line with the National Statement on Ethical Conduct in Human Research (Australian Government, 2007), ethical concerns were addressed prior to ethics approval, and throughout the research process. Respondents were provided with information about the study and were assured of confidentiality of identifiable information and were informed of their right to withdraw participation at any time without prejudice from any party. No incentives were offered.

### Measures

#### Entrepreneurial Self-Efficacy

McGee et al. (2009, p. 966) adopted Bandura’s (1977) characterization of self-efficacy describing it as “an individual’s belief in their personal capability to accomplish a job or a specific set of tasks.” They further examined the underlying dimensionality of ESE drawing on a venture creation model which proposes four discrete phases of the venture creation which they labeled as searching, planning, marshaling and implementing. From their examination, five dimensions of ESE emerged which they categorized as follows: (1) searching, (2) planning, (3) marshaling, (4) implementing-people, and (5) implementing – finance. Their dimensions differ slightly from Chen et al.’s (1998) four-dimension venture creation model with dimensions; (4) people and (5) finance emerging as sub-dimensions of the original implementing dimension. McGee et al.’s (2009) measure is consequently the most comprehensive measure of ESE identified in the extant literature.

Given that the focus of this study is understanding the development of self-employment interest, which occurs before any venture creation has occurred, items related to post-start-up sub-factors were omitted from further analysis (i.e., implementing people and implementing finance).

Pre-venture activities consisting of ten items measuring search, planning and marshaling were then examined. Following a confirmatory factor analysis (refer below) these items were computed into a factor which we titled entrepreneurial self-efficacy- pre-venture (ESE-PV). The alpha reliability for the unidimensional scale was found to be 0.93.

#### Outcome Expectations

A review of the literature failed to identify specific outcome measures that deal with the outcome of being self-employed. A review of the broader career literature located several previously validated measures of outcome expectations scales that were career related. These scales would require tailoring to reflect the context of the present research suitably. The 17-item research outcome expectations scale (ROEQ) in Bieschke (2000) was used as the basis for the customization. Items deemed most fitting with the context of this present research were included in the final measure of outcome expectations. Outcome expectations comprise three forms, incorporating positive and negative physical – material (P), social (SOC), and self-evaluative (SE) outcomes (Bandura, 1986). Consistent with the career literature examining the perceived outcomes of postretirement work (Wöhrmann et al., 2013) two additional items were added related to a perceived positive experience (SE) and financial (P), resulting in a 9-item measure.

Preceding the scale items, it was emphasized that the questions were asking about becoming self-employed. Consistent with the notion that outcome expectations are concerned with imagined consequences of a particular course of action (Lent and Brown, 2006), a common stem was applied before each of the statements included in the scale. The stem read “In general I think starting a business/being self-employed would …” which was followed by the nine items for the measure to complete the stem sentence. An example item is “…enable me to associate with people I value.” Bieschke (2000) reports a Cronbach alpha coefficient range of 0.91 to 0.92 for the original scale. In the present research, the alpha reliability for was scale was found to be 0.93.

#### Interest in Self-Employment

A 5-item scale was adopted to assess individual’s interest in self-employment/business ownership. Following the guidelines of Lent and Brown (2006), participants were asked to what extent they agreed with statements related to self-employment/business ownership. An example is “If I had the opportunity and resources I’d like to start a business/be self-employed.” Internal consistency was 0.96 and above the range usually achieved by reliability for interest scales (i.e., α = 0.75 for engineering activities assessed by Lent and Brown, 2006).

#### Future Time Perspective

Future time perspective was measured using the 10-item FTP scale developed by Carstensen and Lang (Unpublished). Participants rated on a scale from 1 (strongly disagree) to 7 (strongly agree) their level of agreement with the statements. Those with higher scores are deemed to have a more expansive FTP. Three sample items are “Most of my life lies ahead of me,” “I have a sense time is running out,” and “I could do anything I want in the future.” Lang and Carstensen (2002) report a Cronbach alpha of 0.92. In the current study, the alpha coefficient for the scale was found to be 0.92.

#### Support

Perceptions of support for becoming self-employed were measured using a four-item measure adapted from a scale developed by Liñán and Chen (2009) which measures perceived support in terms of whether referent individuals would approve of and support them becoming entrepreneurs. Participants responded on a seven-point Likert-type scale ranging from ‘total disapproval’ to ‘total approval’ and was measured for friends, close family, partner/wife/husband, and colleagues. The items read as “If you were to consider self-employment or starting your own business would [friends, close family, partner/wife/husband, and colleagues] approve/support that decision?” Liñán and Chen (2009) report an internal consistency of 0.77 for the scale. In the current study, the alpha coefficient for the scale was found to be 0.86.

#### Demographic and Control Variables

In addition to the primary variables in the study, two demographic and three additional control variables were measured and included in this research. Control variables were included as the variables have been demonstrated in late-career or entrepreneurship literature to be associated with late-career employment or entrepreneurship behavior – age, gender, education, occupation, length of time with current employer (Virick et al., 2015).

### Analytic Approach

Confirmatory analysis (CFA) was performed to examine the fit of the dimensional models of ESE for the overall sample. McGee et al. (2009) tested three models in the original development of the scale: unidimensional, three-dimensional, and the original five-dimensional model. The indices of model fit considered were the comparative fit index (CFI), root mean square of approximation (RMSEA), the normed χ^2^(χ^2^/df), the Akaike information criterion (AIC), and the root mean squared residual (RMR). A model is considered to have an acceptable fit if the RMR is less than 0.08; the RMSEA close to 0.06 (Hu and Bentler, 1999) or a stringent upper limit of 0.08 (Steiger, 2007); the CFI index is at or above 0.96 (Hooper et al., 2008). Further, a normed χ^2^ lower than 5 suggests a good fit. The AIC is a comparative measure of fit. Lower values indicate a better fit, consequently the model with the lowest AIC is the best fitting model. As we have only considered the three dimensions related to pre-venture activities (searching, panning, and marshaling), a unidimensional and a three-dimensional model were tested against the same fit indices as McGee et al. (2009).

Means, standard deviations, reliability estimates, and bivariate correlations were computed for all variables included in the study using SPSS 25. Next a confirmatory factor analysis, using AMOS 25, was undertaken on the ESE construct to ensure that the five distinct domains (search, plan, marshal, people, and finance) emerged. Following this, the bivariate correlations were examined to check whether relationships existed between the primary variables in the study at a bivariate level.

The PROCESS macro developed by Hayes (2013) was used to test the hypothesized relationships. Analysis was conducted based on the SCCT conceptual framework proposed by Lent et al. (1994). To test the hypothesized relationships, a sequential mediation model was adopted whereby the relationship between the independent variables – IVs (FTP and support) and the dependent variable – DV (interest in self-employment) is sequentially mediated, first by ESE-PV and then by OE. The confidence interval (CI) method for the indirect effect is a bias corrected with acceleration constant for confidence interval estimation (BCa) based on 2000 samples.

## Results

The data collected were screened for assumptions of normality, and missing data, before conducting any analysis.

### Confirmatory Factor Analysis

Table 1 presents the intercorrelations among the three ESE dimensions (search, planning, and marshaling). All bivariate correlations are positive and statistically significant at *p* < 0.01.

**Table 1:** Intercorrelations among the three pre-venture ESE dimensions.

ESE dimension	Planning	Marshaling
Searching	0.784	0.743
Planning	–	0.822

However, although all the correlation coefficients are <1 indicating the absence of complete overlapping between the ESE dimensions, correlation coefficients for searching, planning and marshaling are relatively high from 0.07 to 0.08. A similar result was reported by McGee et al. (2009) and justified the test of the one-dimensional model. Two models where tested – Model 1: the three-dimensional model consisting of searching, planning, and marshaling dimensions; and Model 2 – a unidimensional model resulting combining searching, planning and marshaling. Table 2 shows the results for the CFA of the two models. Model 2 appears to show the best fit. Additionally, Model 2 reported the lowest AIC, indicating it may be the best factorial solution. Furthermore, we tested whether the unidimensional model (Model 2) was significantly better than the three dimensional model (Model 1). Evidence of the prevalence of Model 2 was found (M2 χ^2^ = 48.772, *df* = 28; M1 χ^2^ = 69.980, *df* = 31; Δχ^2^ = 21.208, *df* = 3, *p* < 0.001. Thus, we can conclude that a unidimensional model is the best factorial solution.

**Table 2:** Fit indices for the factorial solutions of the ESE scale.

Groups	CFI	RMSEA	χ^2/df^ Ratio	RMR	AIC
Model 1: Three-dimensional	0.965	0.091	2.257	0.092	117.908
Model 2: Unidimensional	0.981	0.070	1.742	0.073	102.772

*CFI, comparative fic index; RMSEA, root mean square error of approximation; RMR, root mean square residual; AIC, Akaike information criterion*.

### Descriptive Statistics and Correlations

The descriptive statistics (means and standard deviation), scale reliability (Cronbach alpha coefficients) and bivariate correlations among the measured variables are reported in Table 3.

**Table 3:** Means and standard deviation, scale reliability and bivariate correlations among variables.

	Variables	Mean	*SD*	1	2	3	4	5	6	7	8	9	10
(1)	Age	53.18	7.17	–									
(2)	Gender^a^	1.53	0.50	–0.26**	–								
(3)	Education^b^	2.45	1.51	–0.08	–0.19*	–							
(4)	Occupation^c^	1.74	1.09	0.01	0.12	–0.33**	–						
(5)	Time in present job	10.48	10.18	0.24**	0.06	0.02	–0.07	–					
(6)	FTP	4.87	1.19	–0.30**	0.08	0.14	–0.06	–0.06	**0**.**92**				
(7)	Support	5.17	1.21	0.00	–0.05	0.10	–0.00	0.05	0.35**	**0**.**86**			
(8)	ESE-PV	4.84	1.17	0.16	–0.30**	0.34**	–0.13	–0.01	0.34**	0.33**	**0**.**93**		
(9)	Outcome expectations	4.61	1.28	0.11	–0.08	0.06	0.17*	–0.08	0.12	0.26**	0.17*	**0**.**93**	
(10)	Self-employment interest	4.68	1.69	0.15*	–0.16*	0.06	0.10	–0.13	0.24**	0.36**	0.37**	0.65**	**0**.**96**

*^∗∗^Correlation is significant at the 0.01 level (2-tailed). ^∗^Correlation is significant at the 0.05 level (2-tailed). Cronbach’s alpha for the predictor scales on the diagonal.^*a*^Male = 1, Female = 2.*

*^*b*^1 = Secondary School, 2 = Certificate, 3 = Advanced Diploma, 4 = bachelor’s degree, 5 = Graduate Diploma/Graduate Certificate, and 6 = Post Graduate Degree.*

*^*c*^1 = Executive/Manager, 2 = Professional, 3 = Clerical/Administrative worker, 4 = Technicians/trade worker, 5 = sales worker, 6 = Machinery operator/driver, 7 = Community and personal services worker, and 8 = Laborer*.

The FTP was found to be positively and significantly associated with support (*r* = 0.35, *p* < 0.01), ESE-PV (*r* = 0.34, *p* < 0.01) and interest (*r* = 0.24, *p* < 0.01). Support was found to be positively and significantly associated with ESE-PV (*r* = 0.33, *p* < 0.01), OE (*r* = 0.26, *p* < 0.01) and interest (*r* = 0.36, *p* < 0.01). ESE-PV was found to be positively and significantly associated with OE (*r* = 0.17, *p* < 0.05) and interest (*r* = 0.37, *p* < 0.01).

In addition, age was significantly negatively correlated with FTP (*r* = -0.30, *p* < 0.01) and significantly positively correlated with interest (*r* = 0.15, *p* < 0.05). Gender was significantly negatively correlated with ESE-PV and interest (*r* = -0.30, *p* < 0.01, *r* = -0.16, *p* < 0.05, respectively). Education was significantly positively correlated with ESE-PV (*r* = 0.34, *p* < 0.01). Occupation was significantly negatively correlated with ESE-PV (*r* = -0.15, *p* < 0.05).

### Mediation Analysis

The hypotheses were analyzed using the bootstrapping method and PROCESS macro developed by Hayes (2013). To test the hypothesized relationships, a sequential mediation model was adopted whereby the relationship between the IVs (FTP and support) and DV (interest in self-employment) is sequentially mediated, first by ESE-PV and then OE. In addition, while PROCESS does not implicitly permit two IV’s the mediation analysis was conducted in two stages to include both FTP and support as IVs using the following method recommended by Hayes (2013). In the first stage FTP was entered as the IV and support was entered as a covariate. In the second stage support was entered as an IV and FTP was entered as a covariate. The confidence interval (CI) method for the indirect effect is a bias corrected with acceleration constant for confidence interval estimation (BCa) based on 2000 samples.

Age, length of time with current employer, education, gender and occupation were controlled, as they were identified in the literature as being related to the primary variables. Education was significantly related to ESE-PV (*b* = 0.17, *p* < 0.01), indicating that higher levels of education attainment were positively related to ESE-PV. As expected, age was a significant negative predictor of FTP (*b* = -0.05, *p* < 0.01). Occupation was a positive predictor of OE (*b* = 0.32, *p* < 0.001), such that those in lower-level occupations anticipated greater positive OE from self-employment.

**FIGURE 2:**
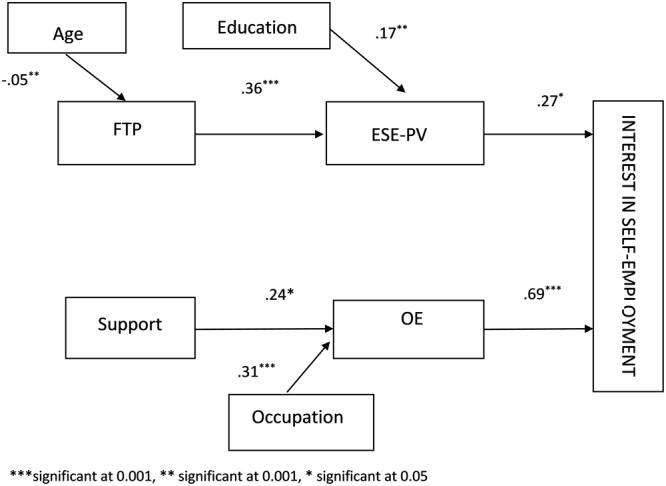
Results of the model of support and FTP as predictors of interest in self-employment mediated by ESE-PV and OE.

The results are summarized in Figure 2. ESE-PV and OE were significant predictors of interest (*b* = 0.27, *p* < 0.05, *b* = 0.69, *p* < 0.001, respectively), supporting hypotheses 1 and 2. Contrary to hypothesis 3, ESE-PV was not significantly related to OE. FTP was significantly related to ESE-PV (*b* = 0.36, *p* < 0.001), supporting hypothesis 4a. Hypothesis 4b indicated that FTP would be related to OE and further, hypothesis 4d predicted OE would mediate the relationship between FTP and interest in self-employment but this was not supported by the results. The relationship between FTP and interest was mediated by ESE-PV [*b* = 0.10, 95%, CI (0.01, 0.23)], supporting hypothesis 4c. Contrary to hypotheses 5a and 5c, support was not significantly related to ESE-PV. Support was significantly related to OE (*b* = 0.24, *p* < 0.05), supporting hypothesis 5b. The relationship between support and interest was mediated by OE [*b* = 0.17, 95%, CI (0.04, 0.32)], supporting hypothesis 5d.

## Discussion

This study contributes to the career and entrepreneurship research in several ways. The findings contribute to our understanding of late career interest in an often overlooked group (older workers), adding to the growing body of recent SCCT research among differing social classes (Flores et al., 2017), race and ethnicities (Ali and Menke, 2014; Dickinson et al., 2017), sexual identities (Tatum et al., 2017) and older workers (Wöhrmann et al., 2013; Garcia et al., 2014). Additionally, the findings support the applicability of SCCT to late career, revealing that older individuals develop late career interests in what they believe they can do and where they anticipated a favorable outcome (Bandura, 1986). Older workers are often contextualized as a homogenous group, with a focus on chronological age. However, the identification of age-specific background and personal factors which influence the development of self-efficacy and outcome expectations for late career suggest that career interest development in later life is complex and multi-faceted. As such, late-career decisions are dynamic and idiosyncratic adding support to the emerging body of career research suggesting that older workers are heterogeneous (Sterns and Miklos, 1995; Bal and Jansen, 2015) and will require individual late career working arrangements that can meet each individual’s motivations and needs.

The findings extend current theory on the complex role of age in the development of self-employment interest in several ways. Overall, the findings confirm the utility of SCCT in the self-employment context and support the model that states FTP and social support predict ESE-PV and OE, which influence the development of self-employment interest for older workers. The model of interest in self-employment for older workers synthesize two historically disparate streams of research investigating career interest and entrepreneurial intentions, in the context of older workers. This novel approach to the examination of self-employment in a career development context provides important insights into the pre-venture, interest development stage and thereby the identification of age-specific barriers and supports to the development of ESE and OE in older individuals. Earlier work on the role of ESE in the entrepreneurial context has focused on it positive influence on entrepreneurial intentions and action. Less research has been devoted to its antecedents.

The inclusion of age-related psychosocial (FTP) and sociocultural (support) factors in the model shed light on the intersection between age (older age), the contextual environment, and development of self-employment interest. The mediating role of ESE-PV and OE adds to our understanding of how interest in self-employment is developed. Prior research on entrepreneurship has tended to overlook the role of OE in the development of entrepreneurial intentions (Chen et al., 1998; Zhao et al., 2005). In the career literature, it is generally argued that OE is principally influenced by self-efficacy (Lent et al., 1994). However, the current findings suggest that OE may be influenced by different personal and background factors than ESE. The dual role of ESE-PV and OE reinforces and expands Bandura’s (1986) arguments that individuals are more likely to develop interest in self-employment when they feel efficacious and expect positive outcomes. The results also provide support for the view that in the case of costly decisions both self-efficacy and outcome expectations influence interest (Lent et al., 1994). For instance, an individual with high self-efficacy for entrepreneurship may not develop an enduring interest if they anticipated a negative outcome (e.g., non-support of referent others, conflict, and financial loss).

Lastly, the identification of FTP as an antecedent to ESE-PV makes an important contribution to the literature examining gray entrepreneurship. Consistent with prior studies examining the age and entrepreneurship (Lévesque and Minniti, 2006) the present study found that age was a significant negative predictor of interest in self-employment. This present finding draws attention to the complex interaction between age and entrepreneurship and reinforces the argument that older workers are a heterogeneous group (Bal and Jansen, 2015). Lastly, the role of social support as an antecedent to OE reinforces the argument that gray entrepreneurship needs external support and approval to be encouraged.

### Practical Contributions

The findings are relevant to practitioners involved in late-career counseling or seeking to nurture interest in self-employment in later life. The results reveal, that despite accumulated knowledge and life experience, support from referent individuals is a salient factor in the development of ESE-PV and OE, for older individuals. Consequently, it is recommended that age-tailored interventions are developed where the aim is to encourage self-employment among older workers. For example, including referent individuals, such as partners and family, in initiatives encouraging older entrepreneurship would be useful. This might include extending entrepreneurial education programs to partners and family. Consequently, interventions increasing the awareness of the positive benefits of entrepreneurship when older may increase individual outcome expectations by garnering support from referent individuals. Utilizing older entrepreneurs as peer mentors can also raise awareness of the positive outcomes from self-employment and increase self-efficacy through role-modeling.

Older individuals with an expanded FTP may be more open to extending their working lives through self-employment as they are more willing to invest in relationships, activities, and goals that have a longer-term return on investment, behaviors which are consistent with early venture creation and development of ESE. Therefore, it would be beneficial to target individuals with an expanded FTP for business start-up programs.

The findings are also relevant to employers. Older workers with an interest in self-employment offer both a risk and an opportunity for organizations. Older workers with strong entrepreneurial interests may retire pre-maturely to pursue their self-employment interests. This may lead to a loss of skills and expertise. It may also lead to a loss of business revenue and missed opportunity if innovative ideas are adopted outside of the business. Retained entrepreneurial employees can be utilized as change agents and innovators who can enhance the organization’s capability (Krueger and Brazeal, 1994). Therefore, organizations seeking to strengthen their entrepreneurial orientation should not overlook older workers. By identifying older workers with a strong ESE-PV, organizations will be able to tap into their entrepreneurial potential and develop opportunities for them to satisfy their entrepreneurial interests in the organization. Likewise, where organizations are seeking to enhance older workers entrepreneurial potential they could develop human resource management initiatives to address age norms related to innovation and entrepreneurial behavior in the organization and provide support networks for older workers to be entrepreneurial.

## Limitations and Future Research

There are several limitations that should be noted considering the present findings. Current generalisability is limited to professional workers. As such, future research could examine the formation of self-employment intentions among non-professional workers such as tradespeople, sales workers and laborers, for example. It also must be acknowledged that not all people will act upon their interest in self-employment. A longitudinal study which examines the transition from interest to goals and finally self-employment (action) would be of interest. Future studies could examine in detail the transition from organizational careers to self-employment and the proximal contextual factors that influence this transition over time.

The originality of using the SCCT framework to study gray entrepreneurial behavior suggests replication of the results is required. Secondly, while this study was precisely designed to assess key dimensions and to control for important factors, further research should examine whether other personal or contextual age-related factors might affect the variables and relationships included in the model.

Despite the practicality of a cross-sectional design for understanding the self-employment interest of older workers, new longitudinal analysis is required to establish stronger causal interpretations are required. For instance, because background contextual factors and ESE and OE were measured at the same this, inferences about temporal ordering of these constructs cannot be assumed. However, the study was designed based on previous theoretical models and existing empirical evidence, suggesting that background contextual factors lead to career self-efficacy and outcome expectations (Lent et al., 1994). Future research could take a longitudinal approach including the examination of the moderating factors theorized by SCCT to influence the relationship between interest and choice goals (Lent et al., 1994).

In conclusion, workforce aging and the need to retain older workers in economic activity beyond what has been normal retirement age, highlight the importance of understanding what factors influence late career choice. Self-employment has become an important career choice for older workers evidenced by the increased uptake of entrepreneurship by people over 45 years of age. This research has contributed to our understanding of the social cognitive antecedents of self-employment interest among older workers. A major practical implication is guidance for organizations and practitioners who are seeking to encourage entrepreneurial interest among older workers and how they can tailor their initiatives.

## Data Availability

The datasets generated for this study are available on request to the corresponding author.

## Author Contributions

VC completed this study as part of a Doctor of Philosophy program. PB supervised the study and revised the writing. JE revised the writing. All authors contributed to manuscript revision, read and approved the submitted version.

## Conflict of Interest Statement

The authors declare that the research was conducted in the absence of any commercial or financial relationships that could be construed as a potential conflict of interest.
