# Corrigendum: Self-Employment in Later Life: How Future Time Perspective and Social Support Influence Self-Employment Interest

**DOI:** 10.3389/fpsyg.2019.02008

**Published:** 2019-08-29

**Authors:** Valerie Caines, Joanne Kaa Earl, Prashant Bordia

**Affiliations:** ^1^Flinders Business, College of Business, Government and Law, Flinders University, Adelaide, SA, Australia; ^2^Department of Psychology, Faculty of Human Sciences, Macquarie University, Sydney, NSW, Australia; ^3^Research School of Management, Australian National University, Canberra, ACT, Australia

**Keywords:** social cognitive career theory, social support, future time perspective, self-employment, older workers, entrepreneurship

In the original article, there was an error. In the “**Results**” section, under “**Mediation Analysis**:” **paragraph 3**, there was a typographical error referencing the relevant hypotheses. A correction has been made so that 5d reads as 5c; and 5c reads as 5d.

The results are summarized in Figure 2. ESE-PV and OE were significant predictors of interest (*b* = 0.27, *p* < 0.05, *b* = 0.69, *p* < 0.001, respectively), supporting hypotheses 1 and 2. Contrary to hypothesis 3, ESE-PV was not significantly related to OE. FTP was significantly related to ESE-PV (*b* = 0.36, *p* < 0.001), supporting hypothesis 4a. Hypothesis 4b indicated that FTP would be related to OE and further, hypothesis 4d predicted OE would mediate the relationship between FTP and interest in self-employment but this was not supported by the results. The relationship between FTP and interest was mediated by ESE-PV [*b* = 0.10, 95%, CI (0.01, 0.23)], supporting hypothesis 4c. Contrary to hypotheses 5a and 5c, support was not significantly related to ESE-PV. Support was significantly related to OE (*b* = 0.24, *p* < 0.05), supporting hypothesis 5b. The relationship between support and interest was mediated by OE [*b* = 0.17, 95%, CI (0.04, 0.32)], supporting hypothesis 5d.

The authors apologize for this error and state that this does not change the scientific conclusions of the article in any way. The original article has been updated.

